# 

**Published:** 1891-02-28

**Authors:** 


In the "Lancet" of Nov. 11, 1865, BARON LIEBIG says:—"Were it possible to furnish the market at a reasonable price with a preparation of meat, combining in itself the albuminous together with the extractive principles, such a preparation would have to be preferred to the Extractum Carnis, for it would contain all the nutritive constituents of meat." Again—"I have before stated that in prein the residue, they are

"BOVRIL" contains know the requirements of the human system and the identical with what the body requires for recuperation, any animal aliment at present known.

paring the Extract of Meat, the Albuminous principles remain lost to nutrition, and this is certainly a great disadvantage."

the albumen and fibrine in the most perfect possible form, and to constituents of food, it will be apparent that this albumen and and that as a perfect form of concentrated nourishment it must

SOLD THROUGHOUT THE UNITED KINGDOM.

### THE HOSPITAL

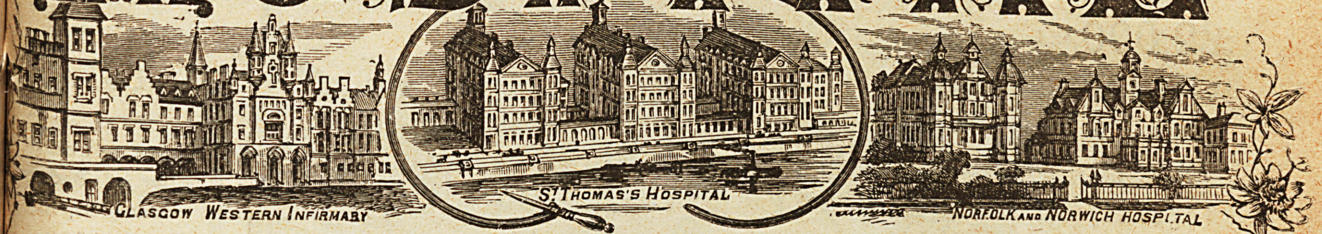

WITH EXTRA NURSING SUPPLEMENT.

No. 231. Vol. IX.] SATURDAY, FEBRUARY 28th, 1891. [ONE PENNY.

